# Vertically Resolved Magma Ocean–Protoatmosphere Evolution: H_2_, H_2_O, CO_2_, CH_4_, CO, O_2_, and N_2_ as Primary Absorbers

**DOI:** 10.1029/2020JE006711

**Published:** 2021-02-23

**Authors:** Tim Lichtenberg, Dan J. Bower, Mark Hammond, Ryan Boukrouche, Patrick Sanan, Shang‐Min Tsai, Raymond T. Pierrehumbert

**Affiliations:** ^1^ Atmospheric, Oceanic and Planetary Physics, Department of Physics University of Oxford Oxford UK; ^2^ Center for Space and Habitability University of Bern Bern Switzerland; ^3^ Department of the Geophysical Sciences University of Chicago Chicago IL USA; ^4^ Institute of Geophysics, Department of Earth Sciences ETH Zurich Zurich Switzerland

**Keywords:** Atmosphere origins, exoplanets, magma oceans, planet composition, planet formation and evolution, planetary surface

## Abstract

The earliest atmospheres of rocky planets originate from extensive volatile release during magma ocean epochs that occur during assembly of the planet. These establish the initial distribution of the major volatile elements between different chemical reservoirs that subsequently evolve via geological cycles. Current theoretical techniques are limited in exploring the anticipated range of compositional and thermal scenarios of early planetary evolution, even though these are of prime importance to aid astronomical inferences on the environmental context and geological history of extrasolar planets. Here, we present a coupled numerical framework that links an evolutionary, vertically resolved model of the planetary silicate mantle with a radiative‐convective model of the atmosphere. Using this method, we investigate the early evolution of idealized Earth‐sized rocky planets with end‐member, clear‐sky atmospheres dominated by either H_2_, H_2_O, CO_2_, CH_4_, CO, O_2_, or N_2_. We find central metrics of early planetary evolution, such as energy gradient, sequence of mantle solidification, surface pressure, or vertical stratification of the atmosphere, to be intimately controlled by the dominant volatile and outgassing history of the planet. Thermal sequences fall into three general classes with increasing cooling timescale: CO, N_2_, and O_2_ with minimal effect, H_2_O, CO_2_, and CH_4_ with intermediate influence, and H_2_ with several orders of magnitude increase in solidification time and atmosphere vertical stratification. Our numerical experiments exemplify the capabilities of the presented modeling framework and link the interior and atmospheric evolution of rocky exoplanets with multiwavelength astronomical observations.

## Key Points

- Magma oceans with different volatiles display varying timescales of solidification, outgassing, and atmospheric stratification
- Atmospheric blanketing and cooling time increase in three principle classes from N_2_, CO, O_2_ to H_2_O, CO_2_, CH_4_ to H_2_
- Coupled mantle–atmosphere systems display distinctive features that link the interior and surface state to atmospheric spectrum and climate

## 1 Introduction

The debate surrounding the formation and long‐term evolution of rocky planets has been dominated by the wealth of data obtained from the terrestrial planets and planetary materials in the Solar System. However, current and upcoming astronomical surveys of planet‐forming circumstellar disks and evolved extrasolar planets will increase the available data greatly. For instance, astrochemical studies of ever‐higher precision deliver clues to the origins of major atmophile elements during the main planet formation era (Bergner et al., 2020; van ’t Hoff et al., 2020). Ongoing (Dreizler et al., 2020; Ricker et al., 2015) and future (Apai et al., 2019; Gaudi et al., 2020; Meixner et al., 2019; Quanz et al., 2019; The LUVOIR Team, 2019) exoplanet surveys will reveal statistically meaningful characteristics of Earth‐sized planets on temperate orbits. Having so far only glimpsed the richness and diversity of the exoplanet census, statistical evaluation of basic physical properties—such as mass, radius, and atmospheric features—teach us that individual rocky planets differ substantially from one another and from the terrestrial planets of the Solar System (Jontof‐Hutter, 2019; Owen, 2019; Pierrehumbert & Hammond, 2019). Shaped by extreme postaccretionary processes, the impinging radiation of close‐in planets can strip away their volatile envelopes, leaving only desiccated, bare rocks (Fulton et al., 2017; Jin et al., 2014; Lopez & Fortney, 2014; Owen & Wu, 2013), with permanent day‐ and nightsides (Kreidberg et al., 2019) or in eternal runaway greenhouse states (Turbet et al., 2019). Further out, being born from volatile‐rich building blocks, solid‐dominated planets may resemble scaled‐up versions of the icy moons of the outer Solar System (Kuchner, 2003; Léger et al., 2004), where high‐pressure ice phases at the mantle‐atmosphere interface inhibit Earth‐like geochemical cycling of nutrients (Journaux et al., 2020; Kite & Ford, 2018; Noack et al., 2017). For the more massive super‐Earths, the crushing pressures at depth generate a supercritical fluid of equilibrated vapor and rock (Kite et al., 2019; Madhusudhan et al., 2020), rendering a surface absent in the traditional sense.

These extreme conditions and processes produce compositional differences between exoplanets that dwarf those between the Solar System terrestrial planets, where from an exoplanet viewpoint the compositional phase space of the Solar System planets is approximately solar (Asplund et al., 2009; McDonough & Sun, 1995) except for the volatile budget (Zahnle & Carlson, 2020). The delivery and redistribution of volatile compounds during planetary evolution constitutes a major gap in our knowledge, yet is crucial for the origin and evolution of terrestrial atmospheres. The uncertainty in volatile behavior limits both our understanding of the planetary environmental context of early Earth and the evolutionary history of rocky exoplanets. Relative to larger, gas or ice‐phase dominated worlds, the surface and climatic conditions of rocky planets are sensitive to a variety of physical and chemical processes that operate during their formation, on their precursors bodies, and after their formation. Planetary embryos are anticipated to undergo orbital migration during the disk phase (Bitsch et al., 2019) and iceline positions evolve (Owen, 2020; Sasselov & Lecar, 2000) during episodic planetesimal formation (Drążkowska & Dullemond, 2018), and thus forming planets can sample an evolving mixture of volatiles in various physical and chemical states during accretion (Eistrup et al., 2018; Öberg et al., 2011). Recent work suggests that forming protoplanets and their precursors experience a significant degree of high‐energy processing (Benedikt et al., 2020; Fegley Jr. et al., 2020; Sossi et al., 2019) during planetary formation. Planetesimals and protoplanets evolve due to impacts (Denman et al., 2020; Kegerreis et al., 2020; Quintana et al., 2016) and internal radiogenic heating (Lichtenberg et al., 2016; Lichtenberg, Golabek, et al., 2019; Lichtenberg, Keller, et al., 2019), both of which dramatically alter the thermal budget and volatile content of young rocky worlds. The composition of early‐ and late‐accreted material can alter the initial oxidation state, and thus chemical speciation of the upper mantle and atmosphere (Gaillard & Scaillet, 2014; Ortenzi et al., 2020; Zahnle et al., 2020), which is relevant for the planetary environment of prebiotic chemistry (Benner et al., 2019; Rimmer & Rugheimer, 2019; Sasselov et al., 2020).

The climatic conditions of young, rocky worlds are therefore established by the extreme cycling of volatiles between planetary reservoirs when the majority of the planet, including its iron core and silicate mantle, is molten. Liquid–gas interactions facilitate rapid chemical equilibration and volatile exchange. The magma ocean phase is thus a crucial evolutionary link between the present state of exoplanets and their formation history (Bonati et al., 2019; Hamano et al., 2015; Lupu et al., 2014; Miller‐Ricci et al., 2009; Schaefer et al., 2016). The abundance of water‐rich phases in carbonaceous chondritic meteorites and the importance of H_2_O as a greenhouse gas motivated pioneering work on steam atmospheres above a magma ocean (Matsui & Abe, 1986a, 1986b; Zahnle et al., 1988), which has since been extended to the early thermal and compositional evolution of young protoplanets (Barth et al., 2020; Bower et al., 2019; Elkins‐Tanton, 2008, 2011, 2012; Hamano et al, 2013, 2015; Katyal et al., 2019; Kite & Barnett, 2020; Lammer et al., 2018; Lebrun et al., 2013; Marcq et al., 2017; Massol et al., 2016; Nikolaou et al., 2019; Salvador et al., 2017; Schaefer et al., 2016; Tikoo & Elkins‐Tanton, 2017). However, meteoritic materials are debris of the specific pathway of the planet formation process in the Solar System (Lichtenberg et al., 2018; Wang et al., 2016) and may differ substantially from the compositional variety in exoplanetary systems. In addition, the compositional deviations between Solar System chondritic groups result in complex transitions in outgassing behavior (Schaefer & Fegley, 2007, 2010), such that the outgassing speciation of chondrite mixtures cannot be deduced from simple superpositions of individual chondrite outgassing speciation (Schaefer & Fegley, 2017). Furthermore, the chemical speciation of protoatmospheres is a result of the dynamic and chemical evolution of the silicate mantle during planet solidification, which acts as a reservoir into which major greenhouse gases partition to different degrees during the magma ocean phase. This sequestering into the interior decouples the evolving climate state of the planet from the volatile speciation of its building blocks.

Evidently, we require advanced tools to assess the planetary state shortly after planetary formation to provide the starting point for the long‐term climatic and geodynamic evolution, and allow us to explore the range of planetary environments that may form. Aiming to further reduce Solar System‐centric assumptions, we here present the evolution and solidification of protoplanets under diverse climatic settings. We focus on major greenhouse gases and compounds that have been suggested to transiently dominate the chemical composition of protoatmospheres. We explore single‐species atmospheres composed of H_2_, H_2_O, CO_2_, CH_4_, CO, O_2_, and N_2_, since these volatiles can become the dominant absorbers under plausible scenarios of planetary accretion and early evolution. For instance, H_2_ can affect the long‐term climate due to collision‐induced absorption (Koll & Cronin, 2019; Pierrehumbert & Gaidos, 2011) and its interaction with N_2_ has been proposed as a contributor to greenhouse warming on early Earth (Wordsworth & Pierrehumbert, 2013a). Furthermore, H_2_ is abundant during planet formation and can create transient reducing climates on young planets. Even though H_2_O is a trace species in the present‐day atmosphere of Earth, it has a strong greenhouse effect which has been routinely discussed in the context of magma ocean protoatmospheres (Abe & Matsui, 1986; Matsui & Abe, 1986a; Zahnle et al., 1988). The often‐remarked influence of H_2_O on the tectonic regime of Earth‐ and super‐Earth‐sized planets suggest that the near‐surface geochemistry is sensitive to its initial abundance (Schaefer & Elkins‐Tanton, 2018; Tikoo & Elkins‐Tanton, 2017)

CO_2_ is another major greenhouse species typically included in models of coupled magma ocean and atmosphere evolution (Bower et al., 2019; Salvador et al., 2017). However, the major phase and partitioning behavior of carbon during planet formation and magma ocean cooling are poorly constrained (Dalou et al., 2017; Fischer et al., 2020; Hirschmann, 2012) despite their relevance for the climate of rocky planets. For instance, CO_2_ can influence the rate of planetary water loss by affecting the cold‐trap temperature and the rate of hydrogen escape (Wordsworth & Pierrehumbert, 2013b), and may be the major factor that determines the past and present climates of Earth or Mars analogs (Forget & Pierrehumbert, 1997; Wordsworth et al., 2017). The dominant species in Earth’s present‐day atmosphere is N_2_, and but its major biogeochemical cycles remain debated (Lammer et al., 2018; Laneuville et al., 2018; Wordsworth, 2016). Specific planetary settings may render CH_4_, CO, and O_2_ the major species in planetary atmospheres for transient epochs. For instance, H_2_‐dominated atmospheres with a mole fraction of H/C of 0.5 may cool down to a CH_4_‐dominated atmosphere at reduced conditions (Hirschmann, 2012). CO‐dominated atmospheres have been suggested as the result of CO capture from extrasolar debris disks (Kral et al., 2020). Finally, O_2_ atmospheres may result from water photolysis and subsequent H_2_ loss in steam atmospheres (Luger & Barnes, 2015; Schaefer et al., 2016; Wordsworth et al., 2018).

Compositional influences on the radiative balance of protoatmospheres will need to be taken into account to probe and refine the potential orbital transition from Venus‐like, primordial runaway greenhouse states, which cool via water dissociation and loss to space, to planets that can cool in an Earth‐like fashion by radiating away primordial heat (Hamano et al., 2013). Long‐lived magma ocean states may be directly observable with near‐future astronomical instrumentation (Bonati et al., 2019; Hamano et al., 2015; Lupu et al., 2014) and link the present‐day climates of extrasolar rocky planets with the ancient climate of Hadean Earth. This would allow crucial insights on the thermal and compositional state of young exoplanets and further help constrain the chronology of terrestrial planets in the Solar System (Fegley et al., 2020). Connecting the thermo‐compositional chronology and histories of exoplanets with observations (Schaefer et al., 2016; Wordsworth et al., 2018) is an essential step to bridge astronomy and planetary science. The goal of this work is thus to demonstrate how a sophisticated coupled model of interior and atmosphere interaction can deliver new understanding in how early climate states are derived from the magma ocean epoch of terrestrial planet evolution. We simulate the energetic and compositional evolution of rocky planets from a fully molten state to their transition to long‐term geodynamic and climatic evolution. We describe our coupled numerical framework in Section 2 and present results in Section 3. We discuss the implications of the work presented here, the main limitations of our approach, and prospects for further studies in Section 4, and conclude in Section 5.

## 2 Methods

**Figure 1:**
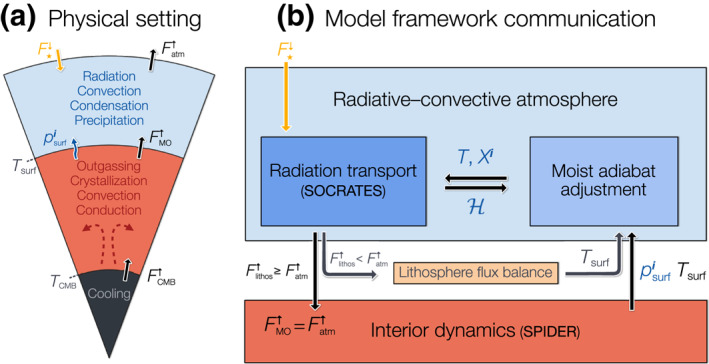
Schematic of the coupled interior–atmosphere model framework. (a) Magma ocean domain with dominant processes and nominal direction of energy fluxes. (b) Computational model internal communication. Arrow directions show information exchange. Boundary conditions for the interior submodule are the temperature at the core–mantle boundary *T*_CMB_, and the net heat loss to space $F_{\text{MO}}^{↑}=F_{\text{atm}}^{↑}$, which is computed by the atmosphere submodule. The interior code computes the boundary conditions for the atmosphere: The surface temperature *T*_surf_, and the surface partial pressure from volatile outgassing $p_{\text{surf}}^{i}$. During late‐stage cooling, the net heat loss of the planet is controlled by the heat flux in the near‐surface (conductive) lithosphere ($F_{\text{lithos}}^{↑}$). In this case, *T*_surf_ is computed by equilibrating lithospheric and atmospheric heat fluxes. The atmospheric profile is constructed iteratively according to the adiabatic lapse rate and the atmospheric heating rate from radiative transfer. The top‐of‐atmosphere boundary is the stellar flux at present time and orbit of the planet.

In order to simulate the thermal evolution of a magma ocean and its outgassing atmosphere, we couple the interior dynamics code spider (Bower et al., 2018, *Simulating Planetary Interior Dynamics with Extreme Rheology*) to the atmosphere radiative transfer code socrates (Edwards & Slingo, 1996, *Suite of Community Radiative Transfer codes based on Edwards and Slingo*). Both codes are stand‐alone and are executed and mediated in a serial loop by a python‐based framework, which synchronizes initial and boundary conditions between the two codes during runtime (Figure 1). The major information exchanged between the interior and the atmosphere is the heat flux and surface temperature at the mantle–atmosphere interface, and the distribution of volatile species between the two reservoirs. We subsequently introduce the individual model components and elucidate how information is communicated between them.

### 2.1 Coupled Atmosphere‐interior Model

#### 2.1.1 Interior

During planetary solidification the rocky planetary mantle undergoes a dynamic transition from a fully molten and turbulent magma ocean to solid‐state convection dominated by viscous creep. In order to capture this evolution, spider (Bower et al., 2018, 2019) follows the interior evolution of an initially fully molten planet during cooling and crystallization by solving the energy conservation equation for a spherically symmetric (1‐D) mantle that is discretized using the finite volume method:

(1) $$\int_{V}\rho T\frac{\partial S}{\partial t}dV=-\int_{A}F_{\text{MO}}^{↑}\cdot dA+\int_{V}\rho HdV,$$

with specific entropy *S*, density *ρ*, temperature *T*, directional heat flux $F_{\text{MO}}^{↑}$, surface area *A*, internal heat generation per unit mass *H*, time *t*, and volume *V*. The heat flux is determined by four dominant energy transport mechanisms that operate in a semi‐molten mantle:

(2) $$F_{\text{MO}}^{↑}=F_{\text{conv}}^{↑}+F_{\text{mix}}^{↑}+F_{\text{cond}}^{↑}+F_{\text{grav}}^{↑},$$

with the directional sum of the individual heat flux contributions from convection $F_{\text{conv}}^{↑}$, latent heat transport by mixing of melt and solid $F_{\text{mix}}^{↑}$, conduction $F_{\text{cond}}^{↑}$, and gravitational separation of melt and solid by permeable flow $F_{\text{grav}}^{↑}$. Note that the individual and total heat flux contributions in Equation 2 can be negative, and thus the model can chart the evolution of both cooling planets ($F_{\text{MO}}^{↑}>0$) and planets that are heated from the top ($F_{\text{MO}}^{↑}<0$) such as the substellar hemisphere of tidally locked super‐Earths that lack an atmosphere. Convection is modeled using mixing length theory by determining a local eddy diffusivity following Abe (1993, 1995, 1997). Compared to 0‐D approaches based on boundary layer theory, mixing length formulations allow for locally determined variations in temperature and composition at all depths in a planetary mantle. The convective heat flux is

(3) $$F_{\text{conv}}^{↑}=-\rho T\kappa_{h}\frac{\partial S}{\partial r},$$

with eddy diffusivity $\kappa_{\text{h}}∼uL_{\text{m}}$, characteristic convective velocity *u*, and mixing length $L_{\text{m}}$. Our nominal model uses a constant mixing length, but we also compute cases where the mixing length is equal to the distance to the nearest mantle interface (i.e., core‐mantle boundary [CMB] or planetary surface) (Stothers & Chin, 1997). Energy is also transported due to the displacement of melt and solid by convection, which causes the release and absorption of latent heat:

(4) $$F_{\text{mix}}^{↑}=-\rho T\Delta S_{\text{fus}}\kappa_{h}\frac{\partial \phi }{\partial r},$$

with entropy of fusion Δ*S*_fus_ = *S*_liq_ − *S*_sol_, liquidus *S*_liq_, solidus *S*_sol_, and silicate melt fraction *ϕ*. Conduction is

(5) $$F_{\text{cond}}^{↑}=-\rho T\kappa_{t}\left(\frac{\partial S}{\partial r}\right)-\rho c\kappa_{t}{\left(\frac{\partial T}{\partial r}\right)}_{S},$$

with thermal diffusivity *κ*_*t*_ and adiabatic temperature gradient (*∂T*/*∂r*)_S_. Finally, molten rock can separate from its surrounding solid rock matrix by permeable flow, and crystals in liquid‐dominated domains can settle or float depending on their density contrast. This exchange of energy is captured by the gravitational separation flux:

(6) $$F_{\text{grav}}^{↑}=a_{\text{g}}^{2}g\rho \left(\rho_{\text{liq}}-\rho_{\text{sol}}\right)\zeta_{\text{grav}}\left(\phi \right)T\Delta S_{\text{fus}}/\eta_{m},$$

with grain size *a*_g_, melt viscosity *η*_m_, density of melt at the liquidus (*ρ*_liq_), density of solid at the solidus (*ρ*_sol_), and flow mechanism factor *ζ*_grav_(*ϕ*). *ζ*_grav_(*ϕ*) parameterizes the flow law as a function of melt fraction, to represent the different regimes from crystal‐bearing suspensions at high melt fraction to porous flow at low melt fraction (see Bower et al., 2018).

We assume the silicate mantle to be isochemical, represented by MgSiO_3_. The thermophysical properties of the pure melt and solid phase are parameterized according to Wolf and Bower (2018) and Mosenfelder et al. (2009), respectively. To derive the material properties for the melt‐solid aggregate we assume volume additivity when computing the two‐phase adiabat. Experiments on synthetic chondritic composition provide the melting curves in the lower mantle (Andrault et al., 2011) and are smoothly joined to the upper mantle melting curves from Hamano et al. (2013), which are based on compiled experimental data on melting of dry peridotite KLB‐1 (Herzberg & Zhang, 1996; Takahashi, 1986; Trønnes & Frost, 2002; Zerr et al., 1998; Zhang & Herzberg, 1994). The melting curves are assumed to be invariant, hence we neglect the potential depression of the solidus by enrichment of volatiles in the residual melt during solidification.

The geophysical (iron) core is not explicitly modeled, but rather a boundary condition is derived at the CMB based on energy balance:

(7) $$\frac{dT_{\text{CMB}}}{dt}=-\frac{4\pi r_{\text{CMB}}^{2}F_{\text{CMB}}}{m_{\text{core}}c_{\text{core}}{\hat{T}}_{\text{core}}},$$

with core mass *m*_core_, core heat capacity *c*_core_, thermal structure correction factor ${\hat{T}}_{\text{core}}=T_{\text{core}}/T_{\text{CMB}}$, mass‐weighted effective temperature of the core *T*_core_, temperature *T*_CMB_, radius *r*_CMB_, and heat flux *F*_CMB_ at the CMB. The boundary condition states that the net thermal energy of the core decreases as a consequence of heat removal from the CMB by the silicate magma ocean. This condition does not account for crystallization of the inner core or heat sources within the core (Chidester et al., 2017). The core is assumed to be isentropic (vigorously convecting), which gives rise to a thermal structure correction factor ${\hat{T}}_{\text{core}}=1.147$. Hence for our initial mantle thermal profile, the initial core temperature *T*_core_ = 5400 K.

#### 2.1.2 Interior–Atmosphere Interface

The cooling rate of a mostly molten magma ocean is limited by the ability of the atmosphere to radiate heat to space. Hence during this stage the flux from the interior (upper boundary condition) is $F_{\text{MO}}^{↑}\equiv F_{\text{atm}}^{↑}$, where $F_{\text{atm}}^{↑}$ is computed by the atmosphere submodule. The surface temperature *T*_surf_ decreases and the mantle cools, driving a redistribution of volatile species *i* between the interior and atmosphere through outgassing. Hence a new surface temperature and distribution of volatiles in the atmosphere is derived, which are then used to update the thermal boundary condition and atmospheric composition of the atmosphere submodule. During the later stages of crystallization, a stiff viscous lid forms once the surface temperature drops below the rheological lockup point such that the interior heat flux can decrease below values that can theoretically be sustained by the atmosphere. Cooling of the interior to space is then inhibited due to the comparatively slow rate of energy transfer through the conductive lid (lithosphere), rather than blanketing by the atmosphere. To account for this we decouple the temperature profile of the lithosphere from the internal profile that is spatially resolved by spider. The surface temperature is then updated by solving for conductive transport in a shallow surface region,

(8) $$\mu_{\text{l}}\frac{dT_{\text{surf}}}{dt}=F_{\text{atm}}^{↑}-F_{\text{lithos}}^{↑}\leq \epsilon ,$$

with the heat capacity of the near‐surface layer *μ*_l_, the lithosphere heat flux $F_{\text{lithos}}^{↑}$ computed from the energy gradient near the surface using spider, and convergence factor *ϵ* = 1 Wm^−2^. The atmosphere is then evolved until it equilibrates with the interior heat flux (Figure 1). When a boundary skin is considered (Figure 9d), we account for the ultra‐thin thermal boundary layer at the surface that offsets the surface temperature from the mantle potential temperature by several hundred Kelvin. The relationship between the boundary layer temperature drop and the surface temperature is derived by equating the heat flux in an ultra‐thin (numerically unresolved) viscous boundary layer with radiation at the surface. For surface temperatures between 1400 K and 2000 K, we then *a priori* solve for the thermal boundary layer structure at steady‐state using the heat flux balance and representative material properties of the melt. This determines that the boundary layer temperature drop scales with the surface temperature cubed with a constant of proportionality of 10^−7^ K^−2^ (Bower et al., 2018; Equation 18).

**Figure 2:**
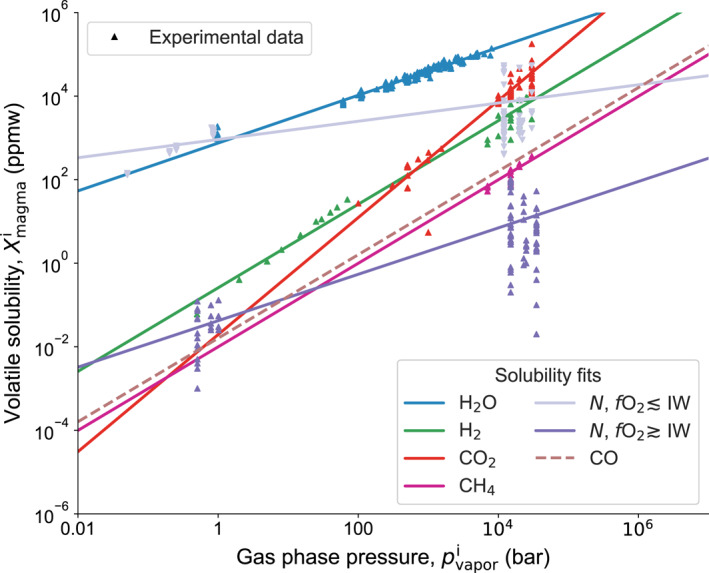
Relationship between ambient pressure and solubility in silicate melts for the considered volatiles from petrological experiments. Data references and fit coefficients *α*_*i*_ and *β*_*i*_ are given in Table 1. Nitrogen solubility is independently calculated where the oxygen fugacity (*f*O_2_) of the magma is below or above the iron‐wüstite buffer (IW), respectively.

A solubility law relates the abundance of a volatile *i* in the interior (in melt) to its abundance in the atmosphere, according to the partial pressure of the volatile at the surface:

(9) $$p_{\text{surf}}^{i}={\left(\frac{X_{\text{magma}}^{i}}{\alpha_{i}}\right)}^{\beta_{i}},$$

with the surface partial pressure $p_{\text{surf}}^{i}$, abundance in the melt $X_{\text{magma}}^{i}$, and Henrian fit coefficients *α*_*i*_ and *β*_*i*_ of species *i* from petrologic data (Table 1, Figure 2). In this compilation we introduce two different relations for nitrogen to illustrate its strongly diverging solubility behavior above and below the iron‐wüstite buffer (Libourel et al., 2003). The chemical composition (as quantified by its redox state, *f*O_2_) of the magma ocean controls the speciation of outgassed volatiles (e.g. Sossi & Fegley, 2018). These chosen relations hence serve to illustrate the solubility behavior for a mantle composition that allows the stability of given volatile species.

**Table 1:** *Partitioning Data and Henry Coefficient Fit Parameters as Shown in Figure*  2

Molecule	*α* (ppmw/Pa)	*β* (nondim.)
H_2_O	1.033 × 10^0^	1.747
H_2_	2.572 × 10^−6^	1.000
CO_2_	1.937 × 10^−9^	0.714
CH_4_	9.937 × 10^−8^	1.000
$N_{2}^{-}$	7.416 × 10^+1^	4.582
N_2_	7.000 × 10^−5^	1.800
CO	1.600 × 10^−7^	1.000
O_2_	–	–

*Note*. Data references—H_2_O: Silver et al. (1990); Holtz et al. (1995); Moore and Carmichael (1998); Yamashita (1999); Gardner et al. (1999); Liu et al. (2005); H_2_: Gaillard et al. (2003); Hirschmann et al. (2012); CO_2_: Mysen et al. (1975); Stolper and Holloway (1988); Pan et al. (1991); Blank et al. (1993); Dixon et al. (1995); CH_4_: Ardia et al. (2013); Keppler and Golabek (2019); N_2_: Libourel et al. (2003); Li et al. (2013); Dalou et al. (2017); Mosenfelder et al. (2019); CO: Fit constants from Yoshioka et al. (2019); O_2_: See main text. $N_{2}^{-}$ indicates the partition coefficients for nitrogen species under reduced conditions with a redox state below the iron‐wüstite buffer (IW), *f*O_2_ ≲ IW

The partial pressure of a volatile $p_{\text{surf}}^{i}$ then relates to the mass of the volatile in the atmosphere (Bower et al., 2019):

(10) $$m_{i}^{g}=4\pi R_{p}^{2}\left(\frac{\mu_{i}}{{\bar{\mu }}_{\text{atm}}}\right)\frac{p_{\text{surf}}^{i}}{g},$$

with planetary radius at the interior–atmosphere interface *R*_*p*_, molar mass *μ*_*i*_ of species *i*, mean atmospheric molar mass ${\bar{\mu }}_{\text{atm}}$, and gravity *g*. We assume O_2_ to be insoluble and thus its atmospheric mass is constant with time. Our treatment of degassing assumes the respective volatile to be in solution equilibrium between the magma ocean and the overlying atmosphere. If outgassing from the magma ocean would be governed by bubble nucleation instead, the degassing rate may decrease (cf. Hamano et al., 2013; Ikoma et al., 2018).

#### 2.1.3 Atmosphere

**Figure 3:**
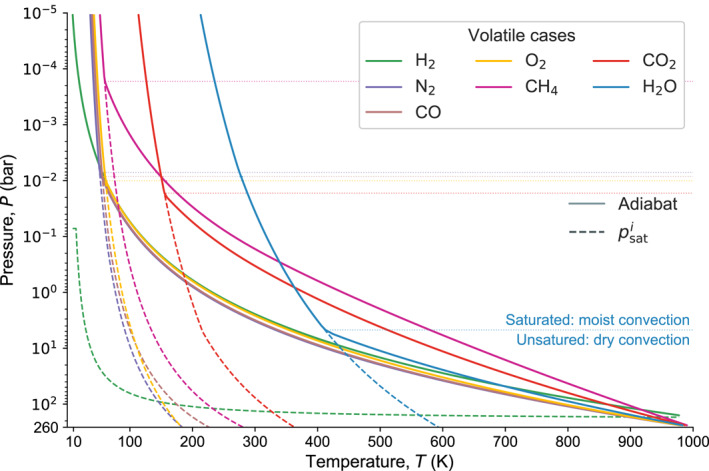
Thermal structure for single species atmospheres with fixed surface pressure equivalent *P*_surf_ = 260 bar and surface temperature *T*_surf_ = 1000 K. In the lower troposphere the atmospheric structure aligns with the dry adiabat until it intersects with the species‐dependent saturation vapor curve, $p_{\text{sat}}^{i}$, at which point condensates form and the lapse rate follows a moist convective profile (Equations 11 and 12).

The atmosphere submodule of the coupled framework establishes the atmospheric thermal and compositional profile, based on the surface partial pressures $p_{\text{surf}}^{\text{i}}$ of the volatile species *i* and surface temperature *T*_surf_. In the earliest, hottest phase, the atmospheric profile follows a dry adiabat, but with cooling the upper atmosphere saturates, condenses, and follows the saturation vapor curve (Pierrehumbert, 2010). Because the net energy flux to space depends on the radiating layer within the atmosphere, it is important to consider the influence of the change in atmospheric lapse rate when temperatures are cool enough for condensation to occur. We use a fourth‐order Runge‐Kutta integrator to compute the atmosphere lapse rate by integrating upwards from the planetary surface where *T* = *T*_surf_:

(11) $$\begin{array}{ccc} \frac{d\ln T}{d\ln P} & =R/c_{p,i}\left(T\right) & if\;unsaturated \end{array}$$

(12) $$\begin{array}{ccc} & =RT/L_{\text{i}} & if\;saturated, \end{array}$$

with universal gas constant *R* = 8.314 JK^−1^ mol^−1^, ambient temperature *T*, and specific heat capacity *c*_*p*,*i*_ and latent heat *L*_i_ per individual species *i*. We treat all gases as ideal to determine thermodynamic properties, such as their critical points and latent heats (Pierrehumbert, 2010). Heat capacities are temperature‐dependent:

(13) $$c_{\text{p,}\;\text{i}}\left(\hat{T}\right)=A_{\text{i}}+B_{\text{i}}\hat{T}+C_{\text{i}}{\hat{T}}^{2}+D_{\text{i}}{\hat{T}}^{3}+E_{\text{i}}/{\hat{T}}^{2},$$

with $\hat{T}=T\left(K\right)/1000$, and the fit constants *A*_i_ – *E*_i_ provided in the NIST Chemistry WebBook, SRD 69 (Chase, 1998; Cox et al., 1984). When condensation occurs during upward integration along the adiabat (Equation 12), a fraction of the gas phase is converted to a condensate phase according to the saturation vapor curve $p_{\text{sat}}^{i}$ of the respective species and removed from the gas parcel (Pierrehumbert, 2010). In the coupled framework, this may occur at ground level indicating formation of an ocean (or glacier; Marcq et al., 2017). The formulation follows a dry adiabat in the lower troposphere, and a moist convective profile once the dry adiabat intersects the Clausius‐Clapeyron slope where condensation occurs. The behavior of the adiabat structure is illustrated in Figure 3 for H_2_, H_2_O, CO_2_, CH_4_, CO, O_2_, and N_2_, with surface pressure *P*_surf_ = 260 bar, surface temperature *T*_surf_ = 1000 K, and top‐of‐atmosphere (TOA) pressure of 10^−5^ bar = 1 Pa.

To account for a potential stratosphere, we iterate between the adiabat adjustment and radiation atmosphere submodules by computing the atmospheric heating rate H (see below). We set the atmosphere to isothermal above the tropopause (Schaefer et al., 2016) and recompute the radiation balance using this adjusted *T*–*P* profile. This procedure yields an approximation to the tropopause location and stratospheric temperature structure and is computationally efficient compared to a time‐stepped radiative equilibrium calculation. To compute the heat flux to space depending on composition and thermal profile of the atmosphere, we utilize the socrates radiative transfer suite (Edwards & Slingo, 1996; Sun, 2011). socrates is incorporated in the UK Met Office Unified Model (Amundsen et al., 2014, 2016) and rocke‐3d (Way et al., 2017), and solves the plane‐parallel, two‐stream approximated radiative transfer equation, which we employ in the limit without scattering. The thermal flux is

(14) $$\pm \frac{1}{D}\frac{dF_{\text{th}}^{↑↓}}{d\tau }=F_{\text{th}}^{↑↓}-\pi B\left(T\right),$$

with thermal upward and downward fluxes, $F_{\text{th}}^{↑}$ and $F_{\text{th}}^{↓}$ respectively, optical depth *τ*, Planck intensity *B*(*T*), temperature *T*, and diffusivity factor *D* = 1.66. Note that *F*, *τ*, and *B* are dependent on wavenumber $\tilde{\nu }$, which we omit here for clarity of notation. The incident stellar radiation is expressed as the direct, unscattered component,

(15) $$F_{⋆}^{↓}=F_{⋆,TOA}\cdot e^{-\tau /\mu }/\mu ,$$

with μ = | cos *θ*| and mean stellar zenith angle *θ* = 54.55°, as a compromise between a daytime‐averaged and insolation‐weighted choice (Cronin, 2014). The surface‐averaged stellar flux at the top of the atmosphere (TOA) is calculated as

(16) $$F_{⋆,TOA}\left(\tau_{⋆}\right)=\frac{1-\alpha_{\text{p}}}{4}\frac{L_{⋆}\left(\tau_{⋆}\right)}{4\pi a_{\text{p}}^{2}},$$

with planetary albedo, bolometric luminosity of the star $L_{⋆}$, and mean orbital distance of the planet *a*_p_. $\tau_{⋆}$ indicates the time since star formation. We assume a constant planetary albedo *α*_p_ = 0.2, which reasonably approximates the albedo over a wide temperature interval of steam atmospheres (Hamano et al., 2015). Varying albedo due to scattering or cloud effects can influence the heat budget of the cooling planet (Pluriel et al., 2019). For the time evolution of the stellar luminosity $L_{⋆}$ we employ a bilinear interpolation in the mass‐time ($M_{⋆}$, $\tau_{⋆}$) plane of the stellar evolution models of Baraffe et al. (1998, 2015) and Girardi et al. (2000). Since we exclusively focus on Sun‐like stars, we use the Solar spectrum from Kurucz (1995). The total upward and downward fluxes are the sum of the thermal and stellar components,

(17) $$F_{\text{net}}^{↑↓}=F_{\text{th}}^{↑↓}+F_{⋆}^{↓}$$

and thus the total atmospheric heat flux of the planet, which is required for the upper boundary condition of the interior, is

(18) $$F_{\text{MO}}^{↑}\equiv F_{\text{atm}}^{↑}=F_{\text{net}}^{↑}-F_{\text{net}}^{↓}.$$

The optical depth at a particular wavenumber in differential form is

(19) $$\begin{array}{ccc} d\tau & = & -k\left(z\right)dz=-k_{\rho }\left(z\right)\rho \left(z\right)dz\\ & = & -\sum \zeta_{i}\left(z\right)k_{\rho }^{i}\left(z\right)\rho \left(z\right)dz, \end{array}$$

with mass mixing ratio *ζ*_*i*_ and mass absorption coefficient $k_{\rho }^{i}$ for volatile species *i*, height *z*, total mass absorption coefficient *k*_*ρ*_(*z*), and total absorption coefficient *k*(*z*) = *k*_*ρ*_(*z*)*ρ*(*z*). The heating rate is then

(20) $$H=-\frac{dF}{dz}=\frac{g{\bar{\mu }}_{\text{atm}}P}{RT}\frac{dF_{\text{atm}}}{dP},$$

with gravitational acceleration *g* and mean molecular weight ${\bar{\mu }}_{\text{atm}}$. socrates achieves computational efficiency by employing the correlated‐*k* method to solve the transmission function: absorption coefficients with similar values are grouped and summed over the atmospheric layers with different weights. *k*‐coefficients are calculated using a combination of correlated‐*k* (Goody et al., 1989) and the exponential sum fitting of transmissions (Wiscombe & Evans, 1977). Line‐by‐line opacity data is banded into *n*_*k*_ subintervals and an average absorption coefficient is calculated from the top of the atmosphere to an optical depth of one,

(21) $$\begin{array}{ccc} k_{\rho ,avg}^{i}\left(\tilde{\nu }\right)u_{\tau =1}^{i} & = & \int_{z_{z=1}}^{\infty }dz^{\prime }\zeta_{i}\left(z^{\prime }\right)\rho \left(z^{\prime }\right)k_{\rho }^{i}\left(\tilde{\nu },z^{\prime }\right)\\ & = & \frac{1}{g}\int_{0}^{P_{\tau =1}}dP^{\prime }\zeta_{i}\left(P^{\prime }\right)k_{\rho }^{i}\left(\nu ,P^{\prime }\right)\equiv 1, \end{array}$$

with column density $u_{\tau =1}^{i}$ from the top of the atmosphere to *τ* = 1 for species *i*. For each subinterval *l* the weighted transmission from line‐by‐line coefficients is fitted to the transmission function, such that

(22) $$\int_{g_{l}}^{g_{l+1}}dg\;w\left(g\right)e^{k_{\rho }\left(g\right)u_{j}}\approx e^{k_{\rho ,opt}^{l}u_{j}}$$

with transmission $e^{k_{\rho ,opt}^{l}u_{j}}$ for a set of *n*_*u*_ column densities, *u*_*j*_, weighting function *w*, and optimal *k*‐coefficient $k_{\rho ,opt}^{l}$ in the given subinterval *l*. Here, we use differential Planckian weights *w*_*l*_ at transmission temperature normalized per band,

(23) $$\int_{0}^{1}dg\;w(g)=\sum_{l=1}^{n_{k}}w_{l}=1.$$

The total flux per wavenumber band *b* is then

(24) $$F_{b}=\sum_{l=1}^{n_{k}}w_{l}^{\tau =1}F_{l},$$

with weights $w_{l}^{\tau =1}$, where the optical depth *τ* = 1 (Amundsen et al., 2014), and flux *F*_*l*_ per subinterval. We use a band structure that covers the wavenumber range from 1–30,000 cm^−1^ and consists of 318 individual bands with a spacing of Δ*ν*_1–120_ = 25 cm^−1^ from 1–3,000 cm^−1^, Δ*ν*_121–280_ = 50 cm^−1^ from 3,000–11,000 cm^−1^, and Δ*ν*_281–318_ = 500 cm^−1^ from 11,000–30,000 cm^−1^. We tabulate the *k*‐coefficients on a *P*, *T* grid of 21 pressures, *P* ∈ ( 10^−11^, 10^−8^, 10^−6^, 10^−5^, 10^−4^, 5 × 10^−4^, 10^−3^, 5 × 10^−3^, 10^−2^, 5 × 10^−2^, 10^−1^, 5 × 10^−1^, 10^0^, 5 × 10^0^, 10^+1^, 5 × 10^+1^, 10^+2^, 5 × 10^+2^, 10^+3^, 5 × 10^+3^, 10^+4^ ) bar, and 15 temperatures, *T* ∈ [ 100, 250, 400, 600, 800, 1000, 1250, 1500, 1750, 2000, 2333, 2666, 3000, 3500, 4000 ] K. The opacity data that is used as input to generate the *k* tables is referenced in Table A1.

## 3 Results

We explore the energetic feedback between a solidifying magma ocean and its outgassed atmosphere. By focusing on single species atmospheres consisting of either H_2_, H_2_O, CO_2_, CH_4_, CO, O_2_, or N_2_, we can isolate the influence of radiative properties and partitioning behavior on planetary cooling timescales. As a baseline for comparing the thermal chronologies of the coupled magma ocean–atmosphere systems, we choose the initial volatile abundance to produce an outgassed atmosphere of 260 bar after mantle solidification. We ignore trapped volatiles in the solidified rock since this abundance is negligible compared to volatile partitioning into the melt. For the case of H_2_O, 260 bar roughly corresponds to a steam atmosphere equivalent of one Earth ocean. All models begin with a fully molten planetary mantle at 100 Myr after star formation.

### 3.1 Steady‐State Atmospheric Radiation Balance

**Figure 4:**
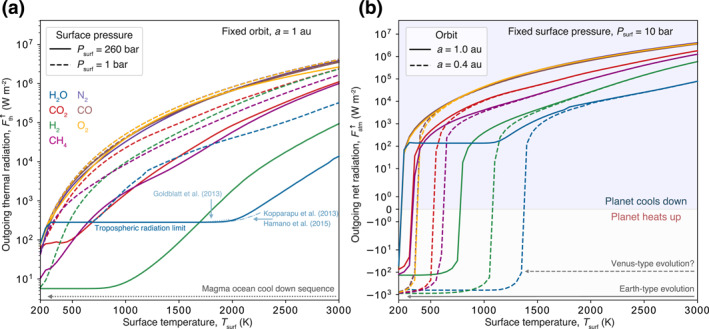
Outgoing radiation for single‐species atmospheres and varying planetary settings. (a) Outgoing thermal radiation as a function of surface temperature for planetary orbital distance *a* = 1 au, and surface pressure equivalent of *P*_surf_ = 260 bar or 1 bar. (b) Outgoing net radiation (thermal minus instellation, Equations 17 and 18) as a function of surface temperature for *a* = 1.0 au or *a* = 0.4 au, with fixed surface pressure of *P*_surf_ = 10 bar and star age $\tau_{⋆}$ = 100 Myr. For closer‐in planets with fixed atmospheric content the incoming stellar radiation exceeds the heat flux to space and they enter the heating regime. The shown results are computed with a fixed planetary albedo of *α*_P_ = 0.2.

Before proceeding to evaluate the evolution of the coupled mantle‐atmosphere system during magma ocean solidification, we illustrate the heat flux properties of the radiative‐convective atmosphere model. We first discuss the steady‐state behavior in order to validate our atmospheric model with previous approaches and illustrate key physical mechanisms that control the heat loss to space. In Figure 4a, we compare the outgoing thermal radiation ($F_{\text{th}}^{↑}$) for all volatiles for surface pressures of 260 bar and 1 bar, which capture the instantaneous state of the atmosphere near the end and start of outgassing, respectively. For the H_2_O–260 bar case we recover the tropospheric radiation limit (Nakajima et al., 1992; Simpson, 1929), with a near‐constant $F_{\text{th}}^{↑}$ of 282.5 Wm^−2^ in the window from ≈ 300–2000 K surface temperature. Our computed radiation limit closely aligns with recent estimates from Goldblatt et al. (2013), Kopparapu et al. (2013), and Hamano et al. (2015) until about 2000 K, and further compares favorably to 280 Wm^−2^ in Marcq et al. (2017) and 282 Wm^−2^ in Katyal et al. (2019). For 260 bar, H_2_ shows a strong depression in the outgoing radiation below the H_2_O radiation limit for *T*_surf_ ≲ 1700 K which appears to be due to its strongly pressure‐dependent opacity. However, validation of the opacity trend at the low temperature range near the surface where H_2_ has high density requires a nonideal H_2_ equation of state. At the lower pressure of 1 bar the radiation limit for H_2_O ranges from *T*_surf_ ≈ 300–700 K and the outgoing radiation for H_2_ is below H_2_O for *T*_surf_ ≲ 450 K. CO_2_ and CH_4_ have similar $F_{\text{th}}^{↑}$ from ≈ 500 K and higher, which is intermediate compared to the lowest $F_{\text{th}}^{↑}$ (H_2_) and the highest $F_{\text{th}}^{↑}$ (O_2_). Relative to the other volatiles, N_2_, CO, and O_2_ do not significantly restrict the heat flux to space. None of the volatiles show strong depression in the outgoing radiation at 1 bar and high temperature.

Figure 4b displays the net outgoing flux ($F_{\text{atm}}^{↑}\equiv F_{\text{MO}}^{↑}$, Equation 18), which serves as the boundary condition at the interior–atmosphere interface when the surface is mostly molten. A significant feature of the 1.0 au cases is the extended radiation limits for H_2_O between *T*_surf_ ≈ 300–1000 K and for H_2_ for *T*_surf_ ≲ 750 K. Once planets are cool enough, their atmospheres become optically thick, and the outgoing radiation becomes smaller than the incoming stellar radiation. This transition occurs below 300 K except for H_2_, so planets at 1.0 au with 10 bar H_2_ atmospheres cannot cool down below ≈750 K in the absence of other processes, such as escape. For closer‐in planets at 0.4 au, the transition points from the cooling to the heating regimes for the various volatiles are separated over a larger range of surface temperature than for planets at 1 au. CO_2_ and CH_4_ show net flux depressions at *T*_surf_ ≲ 500 K, and CO_2_ and CH_4_ at *T*_surf_ ≲ 300 K. The steady‐state calculations reveal the influence of the planetary orbit and atmospheric composition on planetary heat loss, but they do not consider the time variations in both atmospheric volatile budget and thermal state due to progressive solidification of the mantle. The calculations suggest that all volatiles with significant flux depression (H_2_O, H_2_, CO_2_, and CH_4_) may experience a feedback between outgassing and solidification, resulting in marked differences in the cooling timescales. The timescales and atmospheric budgets, however, depend additionally on the solubility of the volatile in the melt. In the next section we thus consider the time‐dependent feedbacks between the interior and atmosphere during the early evolution of planets starting from a fully molten magma ocean.

### 3.2 Time Evolution of the Coupled Magma Ocean–Atmosphere

**Figure 5:**
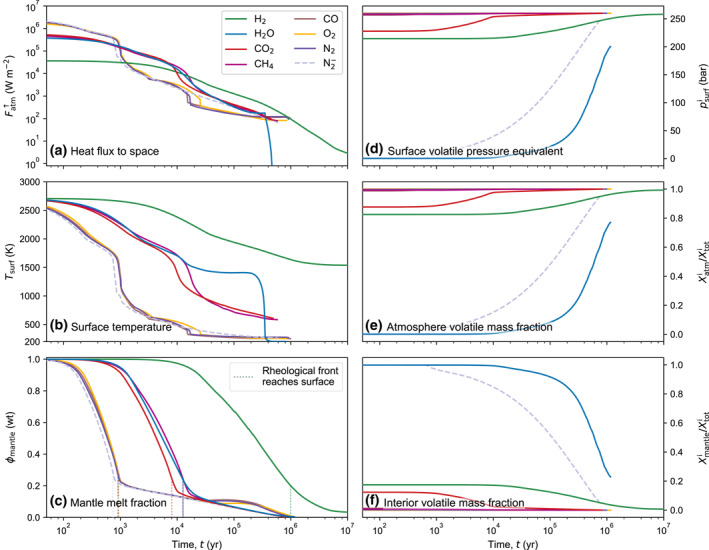
Coupled evolution of mantle and atmosphere. N_2_ is considered for an oxidized (N_2_) and reduced ($N_{2}^{-}$) case. Evolution can be classified into three groups of similar behavior: (a) H_2_, with the most protracted solidification chronology, (b) H_2_O, CO_2_, and CH_4_ with intermediate cooling time, and (c) CO, O_2_, and N_2_/$N_{2}^{-}$ with the shortest cooling time. Times shown are relative to model start, i.e., 100 Myr shifted to star formation. See main text for a more extensive description.

Figure 5 displays the evolution of a solidifying magma ocean for an atmosphere dominated by either H_2_, H_2_O, CO_2_, CH_4_, CO, O_2_, or N_2_ for an Earth‐sized planet at 1.0 au from a Sun‐like star after 100 Myr after star formation. Simulations terminate when the planet reaches a global melt fraction of *ϕ*_mantle_ ≤ 0.5 wt.%. The cooling sequence can be classified into three principal episodes, which vary in duration depending on the volatile species. The first phase is dominated by a fully molten magma ocean (*ϕ*_mantle_ ≈ 1) and varies from ≈10^2^ yr (CO, O_2_, and N_2_) to ≈10^3^ yr (H_2_O, CO_2_, CH_4_), and ≈10^4^ yr (H_2_) in duration. The interior temperatures are superliquidus and the interior convects vigorously which maintains a high surface temperature. During this phase, heat flux, surface temperature, and outgassing of volatiles to the atmosphere vary only minimally. Once the mantle starts to crystallize, the surface temperature decreases faster than during the fully molten phase because heat is not delivered as efficiently to the surface from the deep interior. This is because heat transport is diminished in mixed solid–liquid aggregates or fully solid rock relative to molten magma due to larger viscosity. The second phase occurs when the magma ocean is solidifying from the CMB to the surface. This phase lasts until ≈10^3^ yr for CO, O_2_, N_2_, and ≈10^4^ yr for H_2_O, CO_2_, CH_4_, and ≈10^6^ yr for H_2_. Subsequently the rheological front, which is defined as the (quite abrupt) increase in viscosity from a melt‐ to solid‐dominated aggregate (*ϕ* ≈ 0.4), reaches the surface. This signals a transition to sluggish convection in the interior and hence a protracted cooling phase (phase 3). Solidification proceeds until the mantle reaches near‐zero melt fraction, which takes ≈1 Myr for all volatiles but H_2_. The H_2_ planet case does not solidify within 100 Myr simulation runtime.

The heat flux, surface temperature, and mantle fraction (Figures 5a–5c) result from the feedback between mantle solidification and thermal blanketing by the atmosphere, which limits the heat flux to space depending on the atmospheric conditions. Figures 5d–5f show the outgassing sequence that defines the surface pressure, and total atmospheric and interior volatile mass fraction. The outgassing sequences differ substantially between cases due to the different solubilities of the volatiles in melt (Figure 2). Generally, H_2_O and $N_{2}^{-}$ are the most soluble in magma and hence they outgas late relative to other cases. H_2_O does not outgas completely to establish a 260 bar atmosphere because its solubility is high enough to trap a fraction of H_2_O in the remaining ≈0.5 wt.% of melt. O_2_ is treated as insoluble, and hence its atmospheric mass and surface pressure is constant. For all other cases, outgassing is effectively driven by the reduction in mantle melt fraction, hence outgassing mostly occurs during phase 2 when solidification begins until the rheological front reaches to the surface (at which time ≈ 70–80 wt.% of the planetary mantle is solidified). Outgassing then proceeds until all the volatile mass is transferred to the atmosphere from the interior. Even though volatiles such as H_2_O can be stored in nominally anhydrous minerals, their abundance in the deep mantle is typically around ∼0.1 wt.% (Peslier et al., 2018) which is negligible in comparison to the atmospheric reservoir.

**Figure 6:**
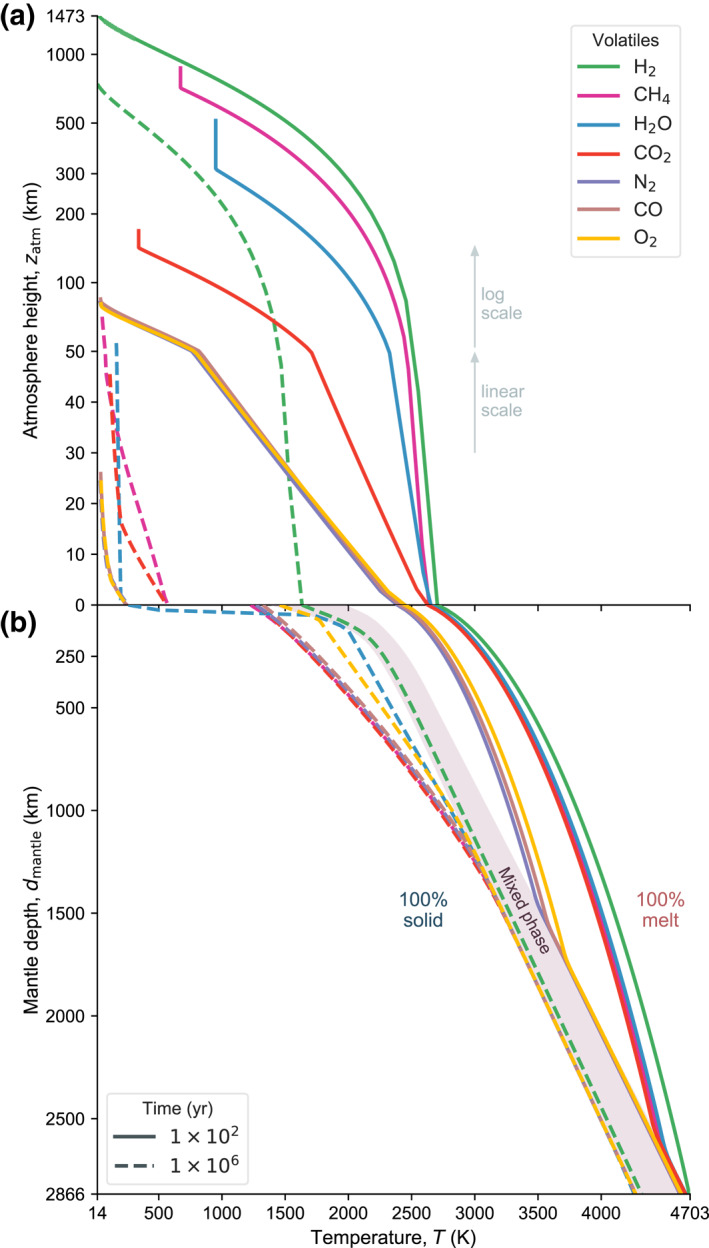
Thermal and vertical structure of the atmosphere and interior at two snapshots during evolution. (a) Atmospheric height stratification. Early atmospheres are thermally expanded and lower molecular weight atmospheres extend to great height. Note the transition in the height axis between linear and log scale at 50 km. (b) Interior evolution. The transition region from fully molten to fully solidified rock is illustrated with a gray background (“Mixed phase”). Right of this area heat transfer is very rapid and dominated by vigorous convection in the liquid magma, left of it heat transfer is much slower and dominated by solid‐state creep processes.

To illustrate the combined evolution of the planet’s interior and atmosphere we show each case at two distinct times in Figure 6. This plot shows the pressure–temperature structure of the atmosphere due to both its composition and radiative‐convective structure. H_2_ displays the most pronounced vertical stratification because of its low molecular weight and slow cooling, followed by CH_4_, H_2_O, CO_2_, then CO, N_2_, and CO. However, at 1 Myr the H_2_O case shows departure of its vertical structure relative to the earlier time and the CH_4_ and CO_2_ cases, and its surface temperature is closer to the ones of CO, O_2_, and N_2_. This emphasizes the influence of the coupled thermal evolution of the atmosphere on the overall planetary structure. Figure 5b reveals the distinct evolution when comparing H_2_O with the CH_4_ and CO_2_ cases. The surface temperature for the H_2_O case is near‐constant between 10^4^–10^6^ yr. However, once the heat flux from the interior drops significantly the surface temperature falls rapidly (Figure 5a) and the atmosphere collapses. This is because for intermediate surface temperatures the atmospheric heat flux remains constant since the upper atmosphere aligns with the H_2_O moist adiabat, i.e., the heat flux is regulated by the radiation limit (Figures 3 and 4). Hence, when the internal heat flux cannot sustain heat loss above the radiation limit anymore, the lithosphere cools rapidly until equilibrium between the internal and atmospheric heat fluxes is reached. Because of the high solubility of H_2_O, most H_2_O outgasses after ≳90 wt.% of the mantle is already solidified and the surface temperature favors condensation. Hence outgassed water immediately forms a condensed surface water reservoir rather than contributing to the atmospheric greenhouse effect. CO_2_ and CH_4_ on the other hand are less effective as absorbers and the planet can cool faster until ≈1 Myr after model start. These volatiles, however, are significantly less soluble in magma compared to H_2_O, and do not facilitate a condensation‐induced radiation limit for temperatures relevant to magma ocean cooling (Figure 4). Therefore, the corresponding planets steadily cool down and their atmospheres grow to their full outgassed 260 bar surface pressure early during magma ocean evolution.

### 3.3 Observational Links Between Atmospheric and Interior State

**Figure 7:**
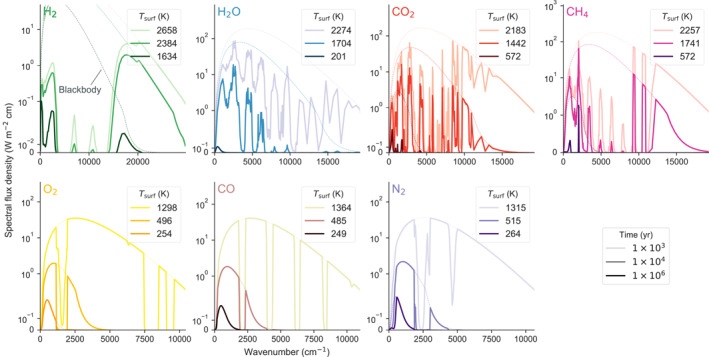
Top‐of‐atmosphere net spectral flux relative to their blackbody spectra for *t* = 10^3^, 10^4^, and 10^6^ yr during magma ocean evolution. These times correspond to when the rheological transition reaches the surface in the different volatile classes (Figure 5).

The sequence of planet solidification for the different volatiles produces time‐evolving atmospheres with different thermal states and spatial extents, which furthermore can provide predictions on the observable spectral signature of the atmosphere. For single‐species atmospheres, the top‐of‐atmosphere emission spectra show distinct features during their evolution and are distinguishable from one another. Figure 7 displays the planetary spectra at three distinct times during evolution when the rheological transition reaches the mantle–atmosphere interface for the different volatile groups at *t* = 10^3^, 10^4^, and 10^6^ yr (Figure 5). The rheological transition denotes when the mode of heat transfer in the mantle transitions from vigorous convection and rapid cooling to more protracted cooling that is dominated by solid‐state convection. In general, higher wavenumber features are muted as the planet solidifies and the spectra across the different absorbers reveal distinct features from each another. H_2_ during early times is dominated by the wavenumber regions around *ν* ≈ 20,000 cm^−1^ but at 1 Myr these regions do not contribute significantly to the heat flux anymore and the planet is dominantly cooling through the regions around *ν* ≲ 3,000 cm^−1^. Similar trends occur for H_2_O, CO_2_, and CH_4_. H_2_O evolves in regions of 4,000–6,000 cm^−1^, CO_2_ in several distinct regions from ≈4,000–15,000 cm^−1^, and CH_4_ at ≈ 3,000 cm^−1^ and between ≈9,000 and 11,000 cm^−1^. CO, N_2_, and O_2_ cool very rapidly and their absorbing regions are confined to ranges around ≈1,500 cm^−1^ for O_2_, ≈2000 cm^−1^ and ≈4,100 cm^−1^ for CO, and ≈2,500 cm^−1^ and ≈ 4,500 cm^−1^ for N_2_. In summary, H_2_, H_2_O, CO_2_, and CH_4_ display the strongest departures from the blackbody curve, which means they absorb more longwave radiation that is emitted from the planetary surface. O_2_, CO, and N_2_ bear significant resemblance to the blackbody curve, so alter the radiation properties of the planet to a lesser degree. Nevertheless, N_2_ displays absorption features around ≈2,500 and ≈ 4,700 cm^−1^ during the fully molten phase.

**Figure 8:**
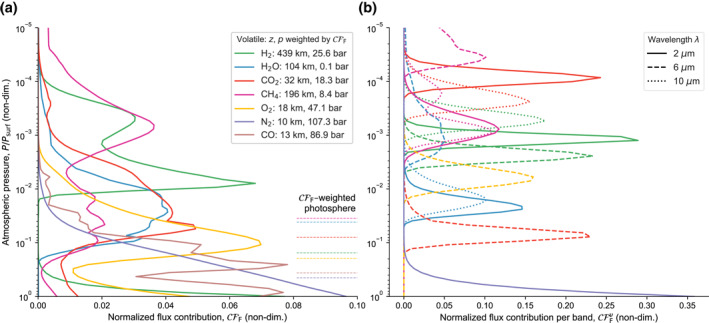
Total and band flux contribution from different atmospheric levels when the rheological front reaches the surface for each simulation (cf. Figure 5). (a) Normalized total flux contribution. Additionally indicated are the ${\mathrm{CF}}_{\text{F}}$–weighted pressure levels of the planets (photosphere). (b) Flux contribution for wavelength bands at *λ* = 2, 6, and 10 μm.

Astronomical observations are typically only possible at confined wavelength regions and thus it is necessary to connect magma oceans with outgassed atmospheres of different compositions to specific features at certain wavelengths. Therefore, Figure 8 compares the contribution of different atmospheric layers to the outgoing flux for the total and wavelength‐specific flux. We quantify the normalized fraction of outgoing net flux that escapes to space from a particular layer *j* in the atmosphere (Drummond et al., 2018):

(25) $${\mathrm{CF}}_{\text{F}}=\frac{2\pi B\left(\tau_{j,j+1}\right)}{D}\left(e^{-D\tau_{j}}-e^{-D\tau_{j+1}}\right).$$

Figure 8a illustrates the contribution of the top‐of‐atmosphere net outgoing flux from different atmospheric layers at different times, and visualizes the dominant photospheres of the magma ocean planets at the transition from melt‐ to solid‐dominated. The flux for the CO, N_2_, and O_2_ cases is dominated by near‐surface layers since these volatiles are least effective as absorbers. In particular for the N_2_ case, the magma ocean surface itself is the dominant emitter, suggesting that planets with N_2_ atmospheres may enable their magma ocean surface to be probed directly. Depending on atmospheric conditions (pressure–temperature), the photosphere shifts to different levels and can sometimes be vertically split, that is, emission can be dominated by two or multiple distinct regions. For instance, the H_2_ case displays two local maxima of the contribution function at ≈ 8 × 10^−3^ and ≈1 normalized pressure. The ${\mathrm{CF}}_{\text{F}}$–weighted pressure level (the photosphere of the planet) lies approximately between these two maxima. The large scale height in Figure 6 suggests that socrates overestimates the opacity of the upper of the two maxima since the height variation of gravity *g*(*z*) is not considered. This could affect the cooling rate by underestimating the mass path, d*m* ∼ d*p*/*g*. For a related discussion for water worlds see Arnscheidt et al. (2019). Therefore, further work is required to estimate the cooling behavior of extended H_2_ atmospheres. The H_2_O and CO_2_ cases mostly emit from intermediate pressure levels between ≈1 × 10^−1^ and ≈5 × 10^−3^ normalized pressure. This analysis demonstrates that we may be able to probe different layers in an atmosphere above a magma ocean (even down to the magma ocean surface) by observing planets with protoatmospheres composed of different dominant volatiles. Wavelengths exhibit different sensitivities with respect to their emitting photospheres. Figure 8b illustrates how the primary emitting surface shifts for 2, 6, and 10 *μ*m. For CO_2_, 2 and 10 *μ*m dominantly probe the upper atmospheric layers, but at 6 μm deep atmospheric layers contribute most significantly to the top‐of‐atmosphere flux. The photosphere depth is not a linear function of wavelength but intimately tied to the opacity of the volatile and the state of the magma ocean and atmosphere. Thus, observations that exploit multiple wavelengths can uncover the thermal stratification with atmospheric depths and reveal deeper or shallower parts of the temperature profile.

**Figure 9:**
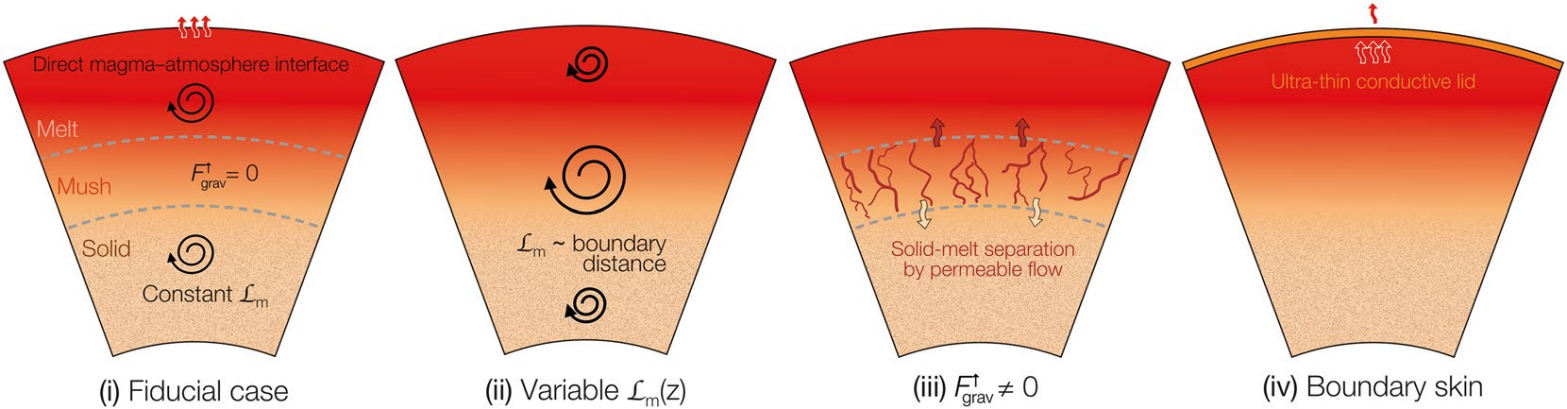
Illustration of interior model parameterizations and influence on mantle solidification. (i) The fiducial case with constant mixing length approximates 0‐D approaches based on boundary layer theory and no separation of melt and solid occurs. Other cases relax an assumption: (ii) Mixing length $L_{\text{m}}$ scales with distance to the nearest domain boundary (magma ocean–atmosphere interface or core‐mantle boundary), meaning that convection is most vigorous in the mid‐mantle and decays toward the boundaries. (iii) Solid and melt can separate via permeable flow. (iv) The magma ocean and atmosphere are separated by an ultra‐thin (∼cm scale) conductive boundary skin, which reduces the surface temperature relative to the interior adiabat thereby increasing the cooling time.

**Figure 10:**
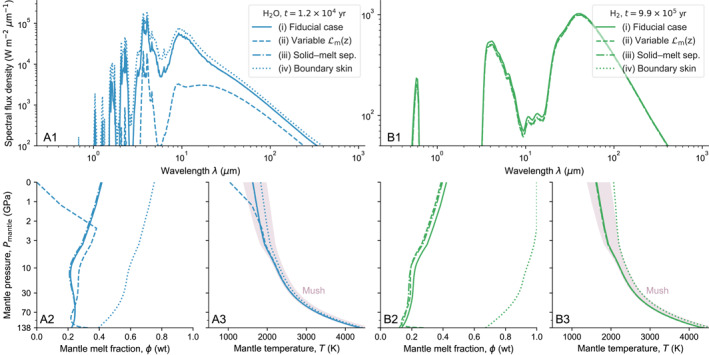
The influence of interior processes on spectral flux density (compare with Figure 9), mantle melt fraction, and mantle temperature for the H_2_O and H_2_ cases when the rheological transition reaches the surface in the fiducial case (Figure 5). (a) H_2_O case at *t* ≈ 10^4^ yr, and (b) H_2_ case at *t* ≈ 10^6^ yr. In A3 and B3, the gray area denotes the mixed region of melt and solid. Note the nonlinear *y*‐axis scale in plots A2, A3 and B2, B3. The boundary skin (iv) delays cooling and modulates the brightness temperature of a largely molten planet relative to the fiducial case.

Figures 9 and 10 explore the sensitivity of the atmospheric state to processes that dictate the evolution of the planetary interior for the H_2_O and H_2_ cases. Figure 9 shows the parameter variations we consider and illustrates how these impact evolution of the interior (see Section 4.3 for discussion). Figure 10 shows how the atmospheric state is perturbed based on processes operating in the interior. In general, spectral flux density variations, relative to the fiducial case, are more apparent for the H_2_O case than the H_2_ case. Varying the mixing length $L_{\text{m}}$ affects the vigor of convection in the mantle and hence leads to the strongest deviations. Solid–melt separation displays no considerable effect and inclusion of a boundary skin at the surface shows constant but small deviations by delaying cooling. An obvious feature for the H_2_O case is the reduced melt fraction at the near‐surface between ≈0 and 2.5 GPa for variable $L_{\text{m}}$ (ii). This melt depletion of the near‐surface comes with decreased surface temperature and thus muting of spectral features, most notably between ≈5 and 20 μm and up to two orders of magnitude at ≈6 μm. At a given time, the boundary skin (iv) leads to strong enhancement of mantle melt fraction relative to the fiducial case by delaying cooling. Thus, the spectrum for this case is shifted relative to the fiducial case at a roughly similar value over all wavelengths. For the H_2_ case, the difference in the spectrum is limited to the variable mixing length. Even though the total mantle melt fraction for the boundary skin case deviates even stronger from the fiducial case (relative to the H_2_O deviation), there is no observable difference in the atmospheric spectrum. This is due to the strong influence of the H_2_ atmosphere, which dominates the cooling rate of the planet (Figure 5). The most notable difference between the H_2_O and H_2_ cases is the lower mantle melt fraction for the variable mixing length parameterization, which is depleted in melt in the H_2_ case. In addition, the H_2_ case with the boundary skin remains above the solidus temperature, such that the upper mantle can sustain a surface magma ocean for an extended duration relative to the fiducial case and the H_2_O case. In short, processes operating in the interior can affect the spectral properties of the overlying atmosphere by up to two orders of magnitude. These are reflected by changes of the spectrum shape, features at specific wavelengths, and whole‐spectrum displacements according to Wien’s law.

## 4 Discussion

Developing the theory and models to connect astrophysical observations to the atmospheric and interior state of terrestrial planets is crucial for elevating our understanding of rocky worlds within and beyond the Solar System. In this work we established a novel modeling framework to investigate the thermal and compositional evolution of young, rocky planets to further remove Solar System bias from considerations of early planetary evolution. In conjunction with astronomical reconnaissance of extrasolar planetary systems, this will allow to untangle the dominant physical and chemical mechanisms shaping the evolution of rocky planets from their formation to their long‐term evolution. To this end, we focused on the radiative effects of varying dominant absorbers during magma ocean solidification.

### 4.1 Atmosphere Formation & Speciation

The bulk composition and eventual climate of evolved planets is a result of the physical and chemical mechanisms that operate during accretion and magma ocean evolution. We presented a modeling framework that vertically resolves the thermal structure of the planet from the core‐mantle boundary at the base of the mantle to the top of the atmosphere. This will allow us in future to investigate vertical variations in mantle and atmospheric chemistry. We focused on the radiative effects of single‐species atmospheres and simulated outgassing and progressing solidification, thereby establishing our coupled modeling approach as a novel tool to explore the early evolution of rocky planets. Our results highlight that various classes of cooling behavior can be expected for different volatiles. However, they do not elucidate the behavior of more complex speciation that may result from changes in the fractionation pattern during volatile delivery. From that perspective, the outgassing behavior of the two contrasting N_2_ cases is particularly relevant, as it highlights the difference between reduced and oxidized planetary interiors. This emphasizes that redox evolution of the planetary mantle and atmospheric speciation are tightly interconnected, as suggested by laboratory experiments (Deng et al., 2020; Grewal et al., 2020). Considering more realistic outgassing speciation as a result of the rock composition (Herbort et al., 2020; Schaefer & Fegley, 2017; Sossi & Fegley, 2018) is thus necessary. In addition, we here ignore the potential effects of dynamic trapping of volatiles due to inefficient melt drainage out of the freezing front during solidification (Hier‐Majumder & Hirschmann, 2017). By altering the outgassing pressure, enhanced volatile trapping may influence the radiating level of the planet, but is mostly confined to the early stages of rapid freezing.

The strong effects of H_2_ on the thermal sequence and crystallization are striking, even with the aforementioned caveat that part of the upper atmosphere opacity may be overestimated in our radiative transfer calculations. To compare the different absorbers using a similar standard, we considered a thick primordial atmosphere that strongly inhibits cooling. We do not consider loss by atmospheric escape (Lammer et al., 2018; Owen, 2019) and thus cannot constrain the competition between outgassing and escape. However, except for the H_2_ case the short magma ocean cooling times do not allow significant time for atmospheric loss. For long‐lived magma oceans, atmospheric escape could play a crucial role during the magma ocean stage of planetary evolution. Recent studies exploring the super‐Earth to mini‐Neptune transition argue for a substantial influence of H_2_ on the mass–radius relation due to its nonideal partitioning into the planetary interior (Chachan & Stevenson, 2018; Kite et al., 2019). Whether or not Earth‐sized planets in temperate orbits can acquire substantial H_2_ atmospheres remains debated, but this could alter the planetary radius if the interior remains molten due to the difference in density between molten and solid rock (Bower et al., 2019). Probing this transition with upcoming space missions will inform whether young rocky planets experience long‐lived phases of melt–solid–atmosphere interaction as we predict for the H_2_ case. This will influence whether the magma ocean stage can completely overprint prior compositional variation inherited during accretion (Lichtenberg, Golabek, et al., 2019).

### 4.2 Geophysical History of Rocky Exoplanets

Upcoming space missions and potentially ground‐based surveys with infrared capabilities may probe the Earth‐sized planetary regime on temperate orbits and will become primary tools to explore the composition and evolution of magma oceans (Bonati et al., 2019; Hamano et al., 2015; Lupu et al., 2014). We illustrate the intimate connection between interior state and atmospheric spectra, and reveal how probing different planets of different compositions can yield insights into interior processes. Planets that are dominated by atmospheric species such as N_2_ or CO, or completely stripped of any atmosphere, may allow the direct observation of the planetary surface. Alternatively, the dependence of the planetary photosphere on composition and wavelength demonstrates that multiwavelength observations may also provide opportunity to probe the temperature structure and atmospheric composition. Observations of magma oceans at different ages can reveal distinct evolutionary stages. Observations of planets around young stars can probe the fully molten stage whereas observations of older planets reveals the sluggish phase of partial solidification; together these establish a chronology of planetary evolution. The short duration of the Earth‐like cooling regime may limit the number of observational targets, depending on the age of the host star and volatile budget and composition (Bonati et al., 2019). However, planets inside the runaway greenhouse limit could offer plenty of targets with long‐lived magma oceans and a wide variety of volatile inventories.

Probing the early stages of planetary evolution is relevant for the history of reduced and oxidized atmospheres. Photolysis and subsequent hydrogen escape could be a main mechanism for water depletion and oxygen build‐up in rocky planetary atmospheres (Luger & Barnes, 2015; Schaefer et al., 2016), and is a popular explanation to interpret exoplanetary observations in the context of planetary evolution and atmospheric composition (Kreidberg et al., 2019). However, our results illustrate that different atmospheric absorbers can produce a variety of thermal and compositional states that evolve as a planet cools, hence setting the stage for prebiotic chemical environments on young planets (Sasselov et al., 2020; Wordsworth, 2012). A natural next step is to incorporate and compare the influence of additional chemical and physical processes, such as atmospheric escape (Odert et al., 2018; Rogers & Owen, 2020; Wordsworth et al., 2018), chemistry (Hu et al., 2012; Pearce et al., 2020), and condensates (Marcq et al., 2017; Pluriel et al., 2019).

### 4.3 Interior State & Tectonics

Generally speaking, we find that for all but H_2_, magma ocean cooling in the Earth‐type regime undergoes similar evolutionary stages as the canonical steam atmosphere: initial rapid cooling during the fully molten phase followed by a phase of more protracted cooling once the mantle reaches the rheological transition. The H_2_ case, however, shows a distinct evolution with comparably protracted cooling and smoothing of the three distinct stages of solidification. In general, the different volatile cases differ by orders of magnitude with respect to the timescale of mantle crystallization. The solidification time has consequences for the compositional stratification of the post‐magma ocean mantle mineralogy and style of solid‐state convection (Boukaré et al., 2015; Elkins‐Tanton, 2008; Solomatov, 2015). Fast cooling, as observed for planets with O_2_, CO, and N_2_ atmospheres, promotes batch crystallization of planetary mantles which decreases the tendency for compositional stratification and large‐scale overturn. In contrast, more protracted cooling, which is favored for H_2_, H_2_O, CO_2_, and CH_4_ cases, promotes fractional crystallization of cumulates and thus whole mantle overturn and compositional stratification.

Furthermore, the solidification time influences the onset of solid‐state convection (Ballmer et al., 2017; Maurice et al., 2017) and the intrusion efficiency of upwelling magma columns, thereby regulating the post‐magma ocean cooling regime of the planet (Lourenço et al., 2018). Tidal effects due to rapid rotation following giant impacts, however, can alter the energetic balance and magmatic evolution of planetary bodies (Lock et al., 2020; Zahnle et al., 2015). From a long‐term geodynamical perspective, mineralogical stratification and potential mantle overturn may result in different styles of mantle convection, separating whole‐mantle convection from double‐layered convection regimes with consequences for long‐term outgassing behavior (Schaefer & Elkins‐Tanton, 2018; Spaargaren et al., 2020). In addition, we directly link the observed spectra of magma ocean planets to interior state by exploring the influence of interior processes on observables. For example, variations in the vigor of magma ocean convection are parameterized by the mixing length parameter, which is analogous to the eddy diffusivity *k*_*zz*_ in atmospheric sciences. Such modeling choices cannot be constrained by observations in the Solar System since magma oceans are no longer present, and laboratory experiments are inherently limited in parameter space. Therefore, astronomical reconnaissance of magma ocean planets may help to further constrain the relative importance of energy transfer processes operating in planetary interiors and inform appropriate parameterizations.

## 5 Summary & Conclusions

We investigated the evolution of the interior and atmosphere of rocky planets during magma ocean solidification with a focus on the radiative effects of protoatmospheres of different composition. The newly established framework of a radiative‐convective atmospheric model coupled to a vertically resolved energy balance model of the planetary mantle allows us to constrain the thermo‐physical evolution of magma ocean planets from the core‐mantle boundary to the top of the atmosphere. We used the radiative‐convective atmosphere model to demonstrate the volatile‐specific planetary cooling rate as a function of surface temperature for fixed atmospheric pressures. We then used the coupled framework to model the time‐dependence of magma ocean solidification for rocky planets dominated by atmospheres comprised of H_2_, H_2_O, CO_2_, CH_4_, CO, O_2_, or N_2_, finding strong variation in their thermal evolution and vertical stratification. We quantified the evolution of their atmospheric spectra and the heat flux contribution from different atmospheric levels during the evolution. This allows inferences of the interior mantle state from atmospheric signals extracted from astronomical observations. Finally, we demonstrated that the relative efficiency of energy transport by different interior processes may have a measurable effect on the associated protoatmospheres. Our coupled magma ocean–atmosphere framework thus expands the solution space in the spatial and compositional domain and makes it possible to investigate planet formation and evolution pathways that differ from the terrestrial planets in the Solar System.

Investigating magma ocean evolution with the coupled model, we find:

- The thermal sequence of magma ocean solidification is influenced by the radiatively dominant volatile to first order; cooling can be protracted by several orders of magnitude for the same orbit and planet configuration. Ordered by increasing timescale of solidification, the volatiles are grouped into three classes:
  − O_2_, N_2_, and CO are least effective as atmospheric absorbers and hence their magma oceans cool the fastest. Because of their low solubilities in silicate melts, their atmospheres form quickly to their full extent and remain at near‐constant surface pressure due to their low condensation temperatures.
  − H_2_O, CO_2_, and CH_4_ establish an intermediate class with more protracted magma ocean cooling. Even though the tropospheric radiation limit for the H_2_O case is most pronounced, CO_2_ and CH_4_ show similar solidification timescales. The reason for this is a combination of their different solubilities in magma, atmospheric extinction efficiencies, and condensation temperatures.
  − H_2_ shows the strongest effect on extending the duration of the magma ocean phase. Even though the evolution still charts the main phases of magma ocean cooling from fully molten to partially solidified to fully solidified, the transitions between these phases are smoothed and delayed relative to the other volatiles. Solidification with an H_2_ atmosphere is not completed within 100 Myr.
- Different volatile cases show different evolution of their atmospheric vertical extent because of the coupled effects of varying volatile solubility, mantle crystallization, and surface condensation.
- Magma ocean protoatmospheres show distinctive features in their spectra for different volatiles and at different times. These may guide future observations to discriminate between atmospheric primary composition and evolutionary stage.
- Spectra are further distinctive depending on interior parameterizations that alter petrological and energetic properties of magma ocean evolution. Therefore, astronomical observations of magma ocean planets may inform further understanding of the geophysical and geochemical evolution of planetary mantles and surfaces.

In conclusion, our work illustrates the importance of coupled models of rocky planetary mantles and their overlying atmospheres to reveal crucial feedback cycles that occur during early planetary evolution. Given the variety of ways to represent the physics and chemistry involved, there is a need for a range of models incorporating various aspects of the problem. We have introduced a novel model that adequately treats the primary energy transport mechanisms operating in a co‐evolving planetary mantle and atmosphere. Future work will build on this modeling framework and pave the way to a more complete understanding of the environmental and surface conditions on young, rocky worlds. We anticipate that further developments—such as laboratory constraints on outgassing, atmospheric chemistry, and cloud feedbacks—will allow to simulate the extensive range of the rocky planet phase space. The insights gained through this venture will enhance our interpretation of the exoplanet census and further constrain the earliest physico‐chemical conditions of our own world.

## Data Availability

The simulation data and plotting scripts to reproduce the presented findings are available at Lichtenberg et al. (2020a); Lichtenberg et al. (2020b, 2020c, osf.io/m4jh7). software: spider (Bower et al., 2018), socrates (Edwards & Slingo, 1996), numpy (Harris et al., 2020; Reddy et al., 2020), scipy (Virtanen, Gommers, Oliphant, et al., 2020; Virtanen, Gommers, Burovski, et al., 2020), pandas (McKinney, 2010; Reback et al., 2019), matplotlib (Caswell et al., 2019; Hunter, 2007), seaborn (Waskom et al., 2018).

## Appendix A Opacity Data

**Table A1:** *List of molecules and references for the opacity data used in this work. Abbreviations: lbl, line‐by‐line; CIA, collision‐induced absorption*

Molecule/system	Type	*ν*_min_ (cm^−1^)	*ν*_max_ (cm^−1^)	Reference
H_2_O	Lbl	0	25,711	Gordon et al. (2017)
H_2_O	continuum			Mlawer et al. (2012)
CO_2_	lbl	158	140,757	Gordon et al. (2017)
CO_2_–CO_2_	CIA	1	2,850	Gruszka and Borysow (1997)
H_2_–H_2_	CIA	20	20,000	Borysow et al. (2001); Borysow (2002)
CH_4_	lbl	0	11,502	Gordon et al. (2017)
CH_4_–CH_4_	CIA	1	990	Borysow and Frommhold (1987)
N_2_	lbl	12	9,354	Gordon et al. (2017)
N_2_–N_2_	CIA	0	5,000	Karman et al. (2015)
O_2_	lbl	0	17,272	Gordon et al. (2017)
O_2_–O_2_	CIA	1,150	29,800	Baranov et al. (2004)
CO	lbl	4	1,447	Gordon et al. (2017)
